# Dysmorphology of inborn errors of metabolism

**DOI:** 10.1186/1755-8166-7-S1-I39

**Published:** 2014-01-21

**Authors:** Virginia Kimonis

**Affiliations:** 1 Division of Genetics and Genomics, Department of Pediatrics, University of California, Irvine, CA 92868, USA

## 

As we discover the molecular mechanism of disorders, eventually all dysmorphic syndromes will ultimately be considered biochemical defects. An overview on the recognition and classification of dysmorphic features will be provided. Categories of inborn errors of metabolism associated with dysmorphic manifestations will be discussed. For e.g. abnormal eye findings are an important clue to the diagnosisin galactosemia, cystinosis, Lowe syndrome, and homocystinuria. Unusual kinky hair is seen in Menkes disease. Skin findings typically lead to the diagnosis in Fabry, Hunter and steroid sulphatase deficiency. Infants who have peroxisomal disorders (Zellweger) pyruvate dehydrogenase deficiency, cholesterol biosynthetic disorders (Smith-Lemli-Opitz), multipleacyl-CoA dehydrogenase deficiency (glutaric aciduria type II), infants of mothers with phenylketonuria have striking facial dysmorphism and structural anomalies at birth. In other disorders dysmorphic features may not be present at birth but may develop with age at varying rates such as in lysosomal storage disorders (mucopolysaccharidoses, oligosaccharidoses), congenital disorders of glycosylation and mitochondrial disorders. Salient clinical features, biochemical defects, molecular basis, and diagnostic strategies will be discussed to permit early treatment of these disorders.

